# On rigid origami III: local rigidity analysis

**DOI:** 10.1098/rspa.2021.0589

**Published:** 2022-02

**Authors:** Zeyuan He, Simon D. Guest

**Affiliations:** Civil Engineering Building, Department of Engineering, 7a JJ Tomson Ave, University of Cambridge, Cambridge CB3 0FA, UK

**Keywords:** foldability, stress, load, first order, prestress stability, second order

## Abstract

In this article, rigid origami is examined from the perspective of rigidity theory. First- and second-order rigidity are defined from local differential analysis of the consistency constraint; while the static rigidity and prestress stability are defined after finding the form of internal force and load. We will show the hierarchical relation among these local rigidities with examples representing different levels. The development of theory here follows the same path as the conventional rigidity theory for bar-joint frameworks, but starts with different high-order rotational constraints. We also bring new interpretation to the internal force and geometric error of constraints associated with energy. Examining the different aspects of the rigidity of origami might give a novel perspective for the development of new folding patterns, or for the design of origami structures where some rigidity is required.

## 1. Introduction

Rigid origami has been developed as a tool for effectively transforming a two-dimensional material into a three-dimensional structure, hence most of the previous studies focus on the kinematics and mechanical properties of foldable rigid origami. In this article, we will consider a different viewpoint—a rigid origami that is not foldable, among which there is a hierarchical relation of different levels of local rigidity. These local rigidity concepts are similar to those used for classical bar-joint frameworks in the structural rigidity theory, but there are also some special features. The basic question of structural rigidity is to ask when a series of length constraints (bars) constrain a number of points (joints) to give a ‘stiff’ structure. Some preliminaries for this question are provided in S1 of the electronic supplementary material. Considering the different aspects of the rigidity of rigid origami might give a novel perspective for the development of new folding patterns, or for the design of origami structures where some rigidity is required. For example, [1,2] analysed the mechanics of periodical rigid origami structures with different levels of local rigidity, inspired from either topological mechanics or Kagome antiferromagnets. We share the same flow in the kinematic analysis despite their rigidity matrix and Hessian having different forms compared to ours.

**Figure 1.**
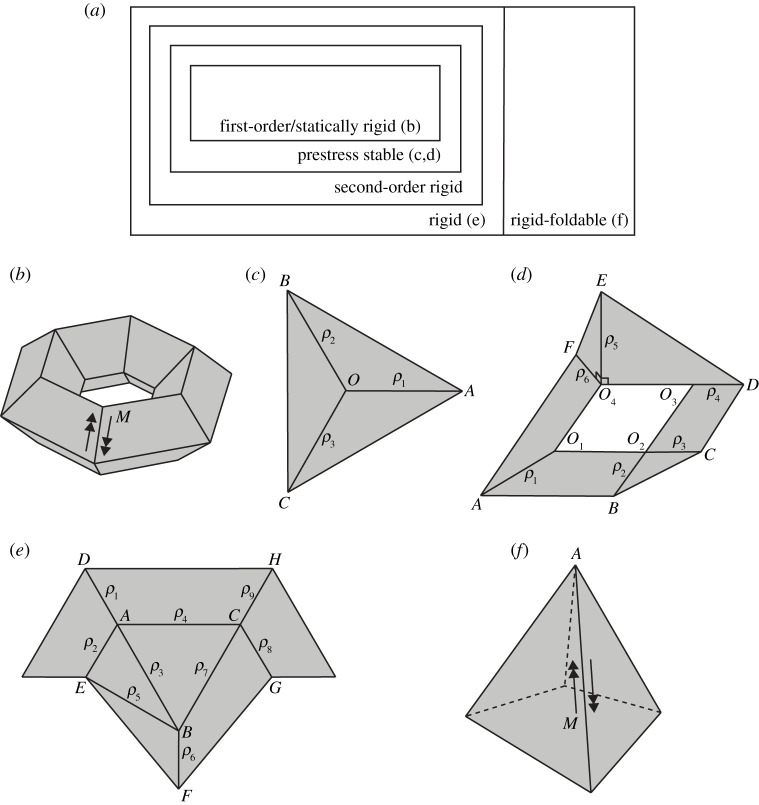
(*a*) The hierarchical relation among different levels of local rigidity for a rigid origami. The first-order rigidity is equivalent to static rigidity, which implies prestress stability, which implies second-order rigidity, which implies rigidity, but none of these relationships is reversible. Panels (*b*–*f*) are examples corresponding to each region in (*a*)—vertices are shown by capital letters, and folding angles by $\rho_{i}$. Panel (*b*) is a 6×4 toroidal polyhedral surface (a handle) that is first-order (equivalently, statically) rigid. Panel (*c*) is a planar degree-3 vertex. Panel (*d*) is a non-planar degree-6 hole, both of which are prestress stable but not first-order rigid. Panel (*e*) is a planar 3-vertex rigid origami that is rigid but not second-order rigid. Panel (*f*) is a rigid-foldable degree-4 vertex. M is a pair of opposite torques applied on two panels incident to an inner crease, which is the form of load for rigid origami. Panel (*b*) is able to carry the load in this configuration, while (*f*) cannot.

The rigid origami model we consider in our series of articles is fairly general, which can be described as rigid panels jointed by hinges without self-intersection, possibly leaving holes (figure 1*d*) and handles (figure 1*b*). The relationship among the local rigidity concepts discussed here, as well as examples of each level of rigidity, are presented in figure 1. The hierarchical relation in figure 1*a* will be proved in §7. The concepts underlying the examples in figure 1*b*–*f* will be presented in the rest of the paper. The 6×4 toroidal polyhedral surface shown in figure 1*b* is first-order rigid (described in §3), i.e. there is no ‘infinitesimal’ motion. The toroidal polyhedral surface is also statically rigid (described in §4), i.e. under any load applied, there would be a set of internal forces generated to keep the rigid origami in equilibrium. Here, the form of load is a pair of opposite torques applied on two panels incident to each inner crease. The example in figure 1*c* is the simplest rigid origami to be prestress stable (described in §5) but not first-order rigid. There is a one-dimensional first-order flex $\left\{\rho_{1}^{\prime },\rho_{2}^{\prime },\rho_{3}^{\prime }\}=a_{1}\{1,1,1\right\}$, $a_{1}\in R$. Although this vertex would be ‘shaky’ along its first-order flex, this configuration will reach a strict local minimum of a predefined potential energy function with a positive self-stress; therefore it will not be able to deform greatly. The example in figure 1*d* has two-dimensional spaces of first-order flex and self-stress, which is also examined to be prestress stable. It turns out that prestress stability is a relatively strong class for common rigid origami structures that are not first-order rigid. The next level is second-order rigidity (described in §6). There is no self-stress that could help the rigid origami reach a local minimum of potential energy, and there is also no first-order flex that can be extended to a second-order flex. Second-order rigidity will imply rigidity. The example in figure 1*e* is rigid but not second-order rigid, which relies on a particular choice of sector angles. The example in figure 1*f* is a degree-4 vertex with centre vertex A where the sum of sector angles is less than 2π, and this vertex is rigid-foldable. Only some loads can be carried. Here, we show a pair of opposite torques that act to change the configuration of this vertex. An interesting point is for some special rigid origami, different levels of rigidity might be equivalent. Such extension of local rigid-foldability is not easy to predict, which will be discussed in §8.

The inspiration for the levels of rigidity comes from classical studies on the statics, prestress stability and second-order rigidity of bar-joint frameworks, e.g. [3–5]. We find a good correspondence between rigid origami and a bar-joint framework. However, rigid origami has some special features. First, because of our kinematic definitions, we do not need to consider the Euclidean motion of a rigid origami in the folding angle description—the only trivial flex is 0, which simplifies some conclusions. Second, in line with classical rigidity theory for a framework, a rigid origami has its special form of the underlying graph and consistency constraint in the folding angle description. For a bar-joint framework, the Jacobian and Hessian of consistency constraints are linear and constant, while for a rigid origami they are in a totally different form of higher order. As a consequence, the form of internal force and load are also different. Third, the effect of self-intersection could not be revealed from the classic method of algebraic analysis on the consistency constraints. The collision between panels might induce rigidity, but this is not considered here—numeric methods are more likely to be efficient when dealing with self-intersection, or even simulating large rotations of a foldable rigid origami. Here, we refer to recent progress on detecting self-intersection [6] and simulating folding motion [7]. Fourth, a set of folding angles might correspond to several stacking sequences (an example is in [8], fig. 2.3), and different stacking sequences might also behave differently when considering the self-intersection of panels, which we also do not consider in this paper. Fifth, when some folding angles are ±π, a flex is valid only when it induces angle change within [−π,π]. Some examples on this topic are provided in S3.6 of the electronic supplementary material. In this article, we will focus on the local algebraic analysis of rigid origami. The effect of self-intersection, stacking sequence and the ‘boundary effect’ when some folding angles are ±π are topics that require further work. In the rest of this paper, we will require every folding angle to be in (−π,π), but it does not mean the conclusions drawn for local rigidity will always fail when some folding angles are ±π.

This paper considers rigidity to the second order. It is natural to ask ‘could this hierarchical relation be extended to countable orders of rigidity and end at finite rigidity?’. However, there seems to exist a limit for such local differential analysis. It turns out that there exists a bar-joint framework that is third-order rigid and flexible [9], which implies the chain relation of local rigidity may not be closed by rigidity, or we might need to modify the definition of local rigidity. Further, for a bar-joint framework, a sufficiently high order flexibility will be equivalent to flexibility; or with extra conditions, they would be equivalent even if the order is not that high [10]. There is a proposal for a revised definition of local rigidity [11], but a complete theory still requires a certain amount of work.

## 2. Consistency constraint on folding angles

In this section we will briefly recap some basic definitions in rigid origami and the consistency constraint on folding angles. The detailed mathematical version is given in [8, Chapter 2], where we provide two roadmaps for the definitions of related concepts: from origami to rigid origami; and from realizations of an underlying graph.

**Figure 2.**
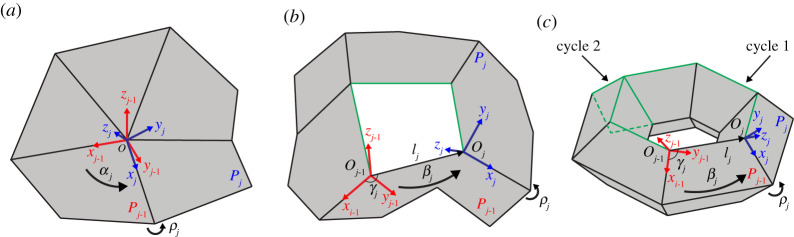
(*a*–*c*) The rotation and possible translation of local coordinate systems around a degree-5 vertex, a degree-5 hole and a representative cycle on a 6×4 toroidal polyhedral surface. The local coordinate systems for $P_{j-1}$ and $P_{j}$ are coloured red and blue, respectively. The representative cycles for (*b*) and (*c*) are coloured green. Specifically, the consistency constraint of (*a*) is around a vertex in the form of equation (2.1); the consistency constraint of (*b*) is around a hole in the form of equation (2.2); the consistency constraint of (*c*) is around 24 vertices and 2 cycles in the form of equations (2.1) and (2.2). (Online version in colour.)

As graphically introduced in figure 2, rigid origami refers to a panel-hinge structure in our series of articles, where finitely many rigid polygonal panels are jointed by line segments (hinges) on their boundaries. We allow contact of different parts of a rigid origami but do not allow crossing, i.e. self-intersection. A panel can only rotate around its adjacent hinges. A rigid origami can have the topology of a sphere with some holes or handles. Line segments on the boundary of panels are called *creases*. The endpoints of creases are called *vertices*. A vertex or crease not on the boundary of a rigid origami is called an *inner*.

Given a rigid origami, we say the angles between adjacent creases on panels are *sector angles*. The range of a sector angle is (0,2π). A *folding angle* at a crease is the difference between π and the dihedral angle measured from a given orientation (a non-orientable rigid origami can always be divided into countable orientable rigid origami that abut along some of their boundaries). The range of a folding angle is [−π,π]. The collections of sector angles and folding angles are written as α and ρ.

Notably, there might be multiple stacking sequences of panels corresponding to the same folding angle ρ. It means that two rigid origami with the same sector and folding angles occupying the same set in $R^{3}$ could have different configurations. Therefore, an *order function* λ is introduced to describe the stacking sequence. The order function λ must satisfy several conditions to prevent self-intersection. Given the sector angles of a rigid origami and lengths of creases of representative cycles (explained below), (**ρ, λ**) can fully describe a configuration of a rigid origami.

Roughly speaking, the distance between two points on a rigid origami is the infimum of lengths of polylines on this rigid origami joining these two points. We say a rigid origami is *flexible*, *foldable* or *rigid-foldable*, or there is a *flex* at (ρ,λ) if there is a continuous family of distance-preserving maps from each time t∈[0,1] to a *rigidly folded state* $\left(\rho_{t},\lambda_{t}\right)$ where $\left(\rho_{0},\lambda_{0})=(\rho ,\lambda \right)$. Otherwise, (ρ,λ) is *rigid*.

Next, we will clarify the constraint on the folding angles ρ. The order function λ is only valid when there is contact between panels and could be examined later. The consistency constraint on folding angles is the collection of consistency condition on each inner vertex and any representative cycles of each hole and handle, which is derived from the rotation and translation of local coordinate systems on each panel near a vertex, a hole and a handle (figure 2). This is the sufficient and necessary condition for a set of folding angles to be an element of the configuration space if allowing the self-intersection of panels.

**Remark 2.1.**

A representative cycle refers to a *homology class* in the *first homology group*, and the number of cycles is called the *first Betti number* [12]. Homology itself was developed as a way to analyse and classify manifolds according to their cycles—closed loops that can be drawn on a given manifold but cannot be continuously deformed into each other. Informally, cycles that can be continuously transformed into each other belong to the same homology class of the first homology group. The first Betti number is also the maximum number of cuts that can be made without dividing a surface into two separate pieces. For example, the first Betti number of a sphere and a disc is 0; of a cylindrical surface it is 1; and of a torus it is 2.

The local coordinate systems are built in the following way when deriving the consistency constraint. (a) Around a vertex surrounded by n panels (figure 2*a*), a *local coordinate system* is built on each panel j (j∈[1,n]), whose origin $O_{j}$ is on the centre vertex, x-axis is on an inner crease, pointing outside the origin, and z-axis is normal to the panel. The direction of all z-axes of local coordinate systems are consistent with the orientation of the paper and hence consistent with the definition of the sign of folding angles. Specifically, the transformation between local coordinate systems of panel j−1 and panel j is a rotation $\alpha_{j}$ along $z_{j-1}$, and a rotation $\rho_{j}$ along $x_{j}$. After a series of rotations, the coordinate system returns to the one built on panel n. The matrix form of transformation is given in equation (2.1). (*b*) Around a cycle surrounded by n panels (figure 2*b*,*c*), we build the local coordinate systems similarly. Each origin $O_{j}$ is on a vertex of this cycle, the x-axis is on an inner crease, pointing outside each origin, and the z-axis is normal to the panel. The transformation between local coordinate systems of panel j−1 and panel j is a translation $\left[l_{j}\cos\gamma_{j};l_{j}\sin\gamma_{j};0\right]$ measured in the coordinate system built on panel j−1, followed by a rotation $\beta_{j}$ along $z_{j-1}$, and a rotation $\rho_{j}$ along $x_{j}$. $\beta_{j}$ and $\gamma_{j}$ can be linearly expressed by the sector angles α. The matrix form is given in equation (2.2).

At every inner vertex $v_{i}\;(1\leq i\leq N_{v}$, figure 2*a*)

2.1 $$R(\rho )=\overset{↷}{\prod_{j=1}^{\deg(v_{i})}}\left[\begin{array}{ccc} \cos\alpha_{j} & -\sin\alpha_{j} & 0\\ \sin\alpha_{j} & \cos\alpha_{j} & 0\\ 0 & 0 & 1 \end{array}\right]\left[\begin{array}{ccc} 1 & 0 & 0\\ 0 & \cos\rho_{j} & -\sin\rho_{j}\\ 0 & \sin\rho_{j} & \cos\rho_{j} \end{array}\right]=I,$$

where $N_{v}$ is the number of inner vertices, $\deg(v_{i})$ is the number of creases (degree) incident to $v_{i}$, $\alpha_{j}$ is between axes $x_{j-1}$ and $x_{j}$ ($2\leq j\leq \deg(v_{i})$), $\alpha_{1}$ is between axes $x_{\deg(v_{i})}$ and $x_{1}$. $\prod^{↷}$ means that in this product of sequence, every new term multiplies from the right-hand side. I is the Identity matrix. Only three of the nine equations are independent, which are in different columns and rows.

At every cycle with boundary $c_{i}\;(1\leq i\leq N_{c}$, figure 2*b*,*c*)

2.2 $$T(\rho )=\overset{↷}{\prod_{j=1}^{\deg(c_{i})}}\left[\begin{array}{cccc} \cos\beta_{j} & -\sin\beta_{j} & 0 & l_{j}\cos\gamma_{j}\\ \sin\beta_{j} & \cos\beta_{j} & 0 & l_{j}\sin\gamma_{j}\\ 0 & 0 & 1 & 0\\ 0 & 0 & 0 & 1 \end{array}\right]\left[\begin{array}{cccc} 1 & 0 & 0 & 0\\ 0 & \cos\rho_{j} & -\sin\rho_{j} & 0\\ 0 & \sin\rho_{j} & \cos\rho_{j} & 0\\ 0 & 0 & 0 & 1 \end{array}\right]=I,$$

where $N_{c}$ is the number of cycles, $\deg(c_{i})$ is the number of creases (degree) incident to $c_{i}$, $\beta_{j}$ is between axes $x_{j-1}$ and $x_{j}$ ($2\leq j\leq \deg(c_{i})$), $\beta_{1}$ is between axes $x_{\deg(c_{i})}$ and $x_{1}$$\times \;[l_{j}\cos\gamma_{j},l_{j}\sin\gamma_{j},0]$ ($1\leq j\leq \deg(c_{i})$) is the position of $O_{j}$ measured in the local coordinate system for panel j−1. $\prod^{↷}$ means that in this product of sequence, every new term multiplies from the right-hand side. I is the Identity matrix. Only six of the sixteen equations are independent. Three of them are in the top left 3×3 rotation matrix; the other three are the elements from row 1 to row 3 in column 4. If the inner creases are concurrent, a cycle will degenerate to a vertex.

If there are $N_{v}$ inner vertices and $N_{c}$ cycles, the number of independent consistency constraints will be $3N_{v}+6N_{c}$. It will simplify further algebraic analysis if we consider the independent components of the consistency constraint. The choice of the particular independent components below will be explained in §3.

For each vertex

2.3 $$\left[\begin{array}{ccc} {}* & * & A_{2}\\ A_{3} & * & *\\ {}* & A_{1} & * \end{array}\right]=R(\rho ).$$

For each cycle

2.4 $$\left[\begin{array}{cccc} {}* & * & A_{2} & A_{4}\\ A_{3} & * & * & A_{5}\\ {}* & A_{1} & * & A_{6}\\ 0 & 0 & 0 & 1 \end{array}\right]=T(\rho ).$$

Here ∗ means elements that are not important for further discussion. A is a vector with length $3N_{v}+6N_{c}$ assembled from three components for each vertex and six components for each cycle.

If ρ is a solution of consistency constraint, A(ρ)=0. However, the converse is not necessarily true: if A(ρ)=0, ρ might not be a solution of the consistency constraint, because equations (2.3) and (2.4) could give rotation matrices whose determinant is 1 but formed by 0 and ±1 apart from the Identity. In other words, the solution space of the independent components of the consistency constraint A(ρ)=0 is larger than the solution space of the consistency constraint. However, these solutions can be easily removed by examination. The first- and second-order derivative of the independent consistency constraint will be used in the analysis of first-order rigidity, prestress stability and second-order rigidity in §§3, 5 and 6.

## 3. First-order rigidity

Given a rigid origami, it is natural to consider possible ‘infinitesimal motion’ allowed by the independent consistency constraint, which leads to the concept of first-order rigidity. The first-order rigidity and first-order flex are defined below. Infinitesimal or first-order motion analysis is also widely applied in all kinds of kinematic analysis.

**Definition 3.1.**

A rigid origami (ρ,λ) is *first-order rigid* if the only solution of $dA/d\rho \cdot \rho^{\prime }=0$ with respect to $\rho^{\prime }$ is 0; equivalently, the rank of *rigidity matrix* dA/dρ equals the number of inner creases $N_{\rho }$. Otherwise, this rigid origami is *first-order rigid-foldable*. A non-zero $\rho^{\prime }$ is called a *first-order flex*, which forms a linear space of dimension $N_{\rho }-\mathrm{rank}(dA/d\rho )$.

We will show how to derive the rigidity matrix dA/dρ for a large rigid origami after writing dA/dρ for its restriction on a vertex or a cycle.

### (a) Rigidity matrix for a vertex or cycle

Consider the first-order derivative of equations (2.3) and (2.4).

3.1 $$\frac{\partial }{\partial \rho_{j}}\left[\begin{array}{ccc} {}* & * & A_{2}\\ A_{3} & * & *\\ {}* & A_{1} & * \end{array}\right]=\left[\begin{array}{ccc} 0 & -x_{3j} & x_{2j}\\ x_{3j} & 0 & -x_{1j}\\ -x_{2j} & x_{1j} & 0 \end{array}\right],$$

where $x_{j}=[x_{1j};x_{2j};x_{3j}]$ is the direction (column) vector of the inner crease $\rho_{j}$ measured in a global coordinate system, pointing away from this vertex.

3.2 $$\frac{\partial }{\partial \rho_{j}}\left[\begin{array}{cccc} {}* & * & A_{2} & A_{4}\\ A_{3} & * & * & A_{5}\\ {}* & A_{1} & * & A_{6}\\ 0 & 0 & 0 & 1 \end{array}\right]=\left[\begin{array}{cccc} 0 & -x_{3j} & x_{2j} & \\ x_{3j} & 0 & -x_{1j} & O_{j}\times x_{j}\\ -x_{2j} & x_{1j} & 0 & \\ 0 & 0 & 0 & 1 \end{array}\right],$$

where $x_{j}=[x_{1j};x_{2j};x_{3j}]$ is the direction (column) vector of the inner crease $\rho_{j}$ measured in a global coordinate system, pointing away from this cycle. $O_{j}$ is the position of vertex on the cycle incident to $\rho_{j}$ measured in the global coordinate system (figure 2). × means the cross product of two vectors. The derivation is provided in S2 of the electronic supplementary material. This result has previously been presented in [18,19].

The reason for choosing the particular components when defining the independent consistency constraint A(ρ)=0 is to ensure that we capture independent non-zero values in equations (3.1) and (3.2) to describe the ‘speed’ of a dynamic system.

The matrix form of rigidity matrix for a degree-n vertex or a cycle could therefore be written as

3.3 $${\frac{dA}{d\rho }}_{\text{vertex}}=\left[\begin{array}{cccc} x_{1} & x_{2} & \cdots & x_{n} \end{array}\right]$$

and

3.4 $${\frac{dA}{d\rho }}_{\text{cycle}}=\left[\begin{array}{cccc} x_{1} & x_{2} & \cdots & x_{n}\\ O_{1}\times x_{1} & O_{2}\times x_{2} & \cdots & O_{n}\times x_{n} \end{array}\right].$$

The rigidity matrix dA/dρ for each vertex or cycle can also be explained from analytical mechanics. First, around each degree-n vertex, the virtual rotation from panel n to panel j induced by a perturbation on folding angles δρ is $x_{1}\delta \rho_{1}+x_{2}\delta \rho_{2}+\cdots +x_{j}\delta \rho_{j}$. After returning to panel n, the relative virtual rotation should be 0, therefore,

3.5 $$\sum_{1}^{n}x_{j}\delta \rho_{j}=0.$$

Around each degree-n cycle, equation (3.5) still holds. Fix panel n to exclude Euclidean motion, the virtual displacement from origin of the global coordinate system 0 to the xy-plane of local coordinate system built on panel j induced by a perturbation on folding angles δρ is $O_{1}\times x_{1}\delta \rho_{1}+O_{2}\times x_{2}\delta \rho_{2}+\cdots +O_{j}\times x_{j}\delta \rho_{j}$. For panel n, this virtual displacement should be 0, hence around each cycle we have

3.6 $$\sum_{1}^{n}(O_{j}\times x_{j})\delta \rho_{j}=0.$$

### (b) Measure of deformation about a vertex or cycle

Given a rigid origami (ρ,λ), it is instructive to consider the form of the deformations that we are not allowing if there is a perturbation on folding angles δρ. A measure of deformation called *first-order error* ε(ρ) could be derived from the first-order estimation of the independent consistency constraint A(ρ+δρ)

3.7 $$\varepsilon (\rho )=\frac{dA}{d\rho }\delta \rho .$$

For a degree-n vertex

3.8 $$\left[\begin{array}{c} A_{1}\\ A_{2}\\ A_{3} \end{array}\right]=\left[\begin{array}{c} \varepsilon_{1}\\ \varepsilon_{2}\\ \varepsilon_{3} \end{array}\right]+O(\delta \rho^{2})=\sum_{1}^{n}x_{j}\delta \rho_{j}+O(\delta \rho^{2}).$$

Here, the first-order error is the components about the global x, y and z axes of the rotation from the local coordinate system built on panel n to itself, as a circuit is taken around the vertex with folding angles ρ+δρ.

For a degree-n cycle

3.9 $$\left[\begin{array}{c} A_{1}\\ A_{2}\\ A_{3}\\ A_{4}\\ A_{5}\\ A_{6} \end{array}\right]=\left[\begin{array}{c} \varepsilon_{1}\\ \varepsilon_{2}\\ \varepsilon_{3}\\ \varepsilon_{4}\\ \varepsilon_{5}\\ \varepsilon_{6} \end{array}\right]+O(\delta \rho^{2})=\left[\begin{array}{c} \sum_{1}^{n}x_{j}\delta \rho_{j}\\ \sum_{1}^{n}O_{j}\times x_{j}\delta \rho_{j} \end{array}\right]+O(\delta \rho^{2}).$$

The first-order error is the rotation described above, and the change of signed distance from origin of the global coordinate system 0 to the xy-plane of local coordinate system built on panel n, as a circuit, is taken around the boundary of the cycle with folding angles ρ+δρ.

**Figure 3.**
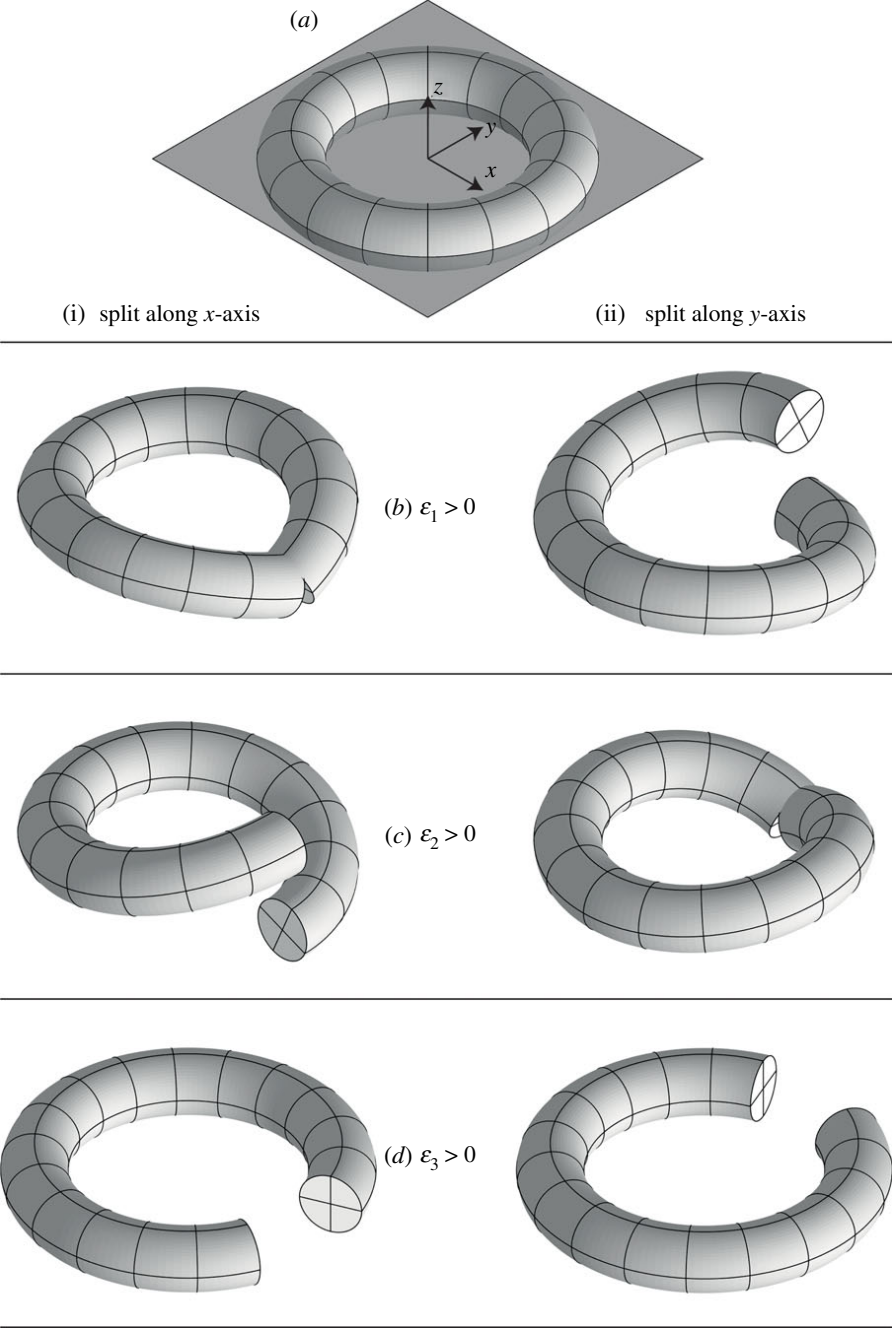
An illustration of the first-order error ε in a global coordinate system around a vertex. Here, we use a cut torus to show the unit first-order error of the constraint around a vertex in an elastic view, and we point out these first-order errors correspond with unit rotation around hinges that are parallel to the direction of axes. Note that there is a fundamental difficulty in showing this deformation, as it is a measure of what rigid origami *cannot* do, so a cut has to be introduced to allow the deformation to be shown, and where the cut is introduced *may* make a big difference to the appearance. (*a*) We attach a torus to the paper around the vertex. (*b*) We first consider a deformation $\varepsilon_{1}$ distributed evenly around the torus, so that there is a constant ‘curvature’ around the x-axis (which in places manifests as a twist). The torus is then not able to close, so we show the deformed torus with a cut along the (i) x-axis, or (ii) y-axis. Although the images look very different, the underlying deformation is the same in each case. (*c*,*d*) We similarly show the deformation $\varepsilon_{2}$ and $\varepsilon_{3}$. Only for (*b*)(i) and (*c*)(ii) would a first-order error be compatible with the rotation of a hinge, in x- and y-directions, respectively. The internal forces that are work-conjugate with these first-order errors are shown in figure 4.

For a vertex, a graphical representation of first-order errors $\varepsilon_{1},\varepsilon_{2},\varepsilon_{3}$ is provided in figure 3. The consistency constraint is illustrated by a closed torus. When there is a cut, the constraint is released, and the first-order errors are shown by the rotation of cross-section of the torus. Suppose n=1, a first-order error ε would be a rotation $\delta \rho_{1}$ along direction $x_{1}$; in figure 3*b*(i) and figure 3*c*(ii), we could see that a positive first-order error when $\delta \rho_{1}>0$ is compatible with a positive folding angle if considering the cut as rotation of panels around an inner crease.

### (c) Rigidity matrix for a large rigid origami

Now we consider assembling the derivative for each vertex or cycle in equations (3.3) and (3.4) to a large rigid origami. In view of the programming, information about the crease pattern could be stored in a *incidence matrix* D describing the relationship between inner creases and vertices with a labelling of them. If vertex i is incident to inner crease j and the direction vector goes out from i, $D_{ij}=1$; if the direction goes towards i, $D_{ij}=-1$, otherwise $D_{ij}=0$. D is a sparse matrix. An example is provided in S3.1 of the electronic supplementary material.

## 4. Static rigidity

We will now consider the behaviour of a rigid origami when load is applied. First, we will introduce a restricted set of external applied loads and internal forces that are work-conjugate to the kinematic quantities mentioned in the previous section, before pointing out how these might be related to more general sets of forces.

The equilibrium analysis starts from the principle of virtual work. A virtual displacement for rigid origami is exactly an arbitrarily small first-order flex $\rho^{\prime }$ at a rigidly folded state (ρ,λ). We define a *load* l so that the external virtual work done by the load $\delta W_{e}$, for any $\rho^{\prime }$, is given by

4.1 $$\delta W_{e}=l\rho^{\prime }.$$

Thus the form of the load must be a set of equal and opposite torques applied to the panels on each side of each inner crease, such that positive external virtual work is done by a positive change in folding angle at the crease (remembering that a valley fold corresponds with the positive direction of folding angle).

Consider also the internal forces that may exist within the rigid panels. We define the *internal forces* ω such that the internal work done $\delta W_{i}$, for any first-order error $\varepsilon (\rho^{\prime })$, is given by

4.2 $$\delta W_{i}=\omega \varepsilon (\rho^{\prime })=\omega \frac{dA}{d\rho }\rho^{\prime }.$$

**Figure 4.**
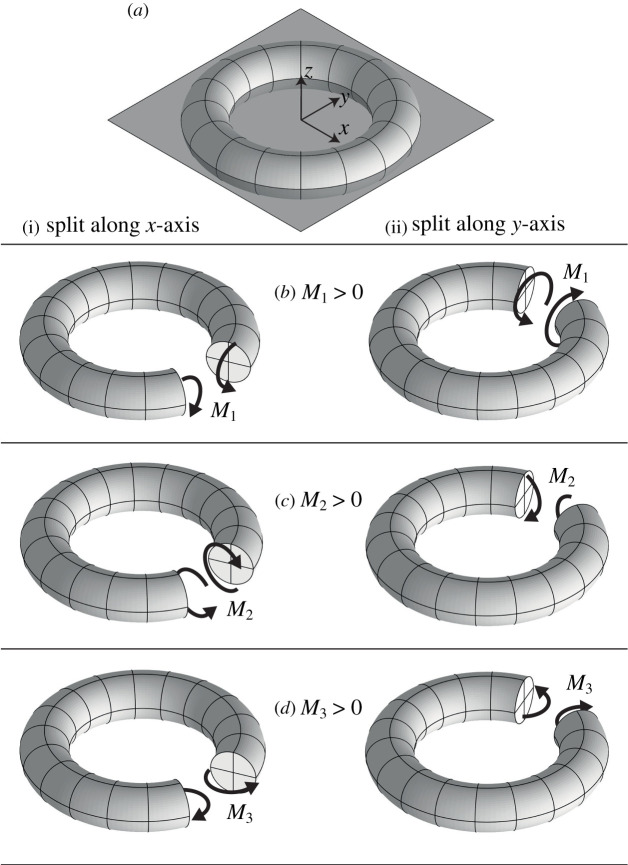
The internal forces (here, torques) $\left\{M_{1},M_{2},M_{3}\right\}$ that are work-conjugate to the first-order errors $\left\{\varepsilon_{1},\varepsilon_{2},\varepsilon_{3}\right\}$ distributed around a torus embedded in a paper, as introduced in figure 3. In each case, the internal force is only shown where there is a cut. Only in (*b*)(i) and (*c*)(ii) can these internal forces be directly applied by a pair of opposite torques at a hinge (along the x- and y-axes, respectively). The deformations shown in figure 3 are not, *in general*, elastic responses to the forces shown here, although in fact they would correspond to the elastic response of a cut torus that had equal bending and torsional stiffness.

Thus the form of the internal force should be an internal torque around each inner vertex, and an internal torque and force around each cycle. For a vertex, a graphical representation of the internal force ω is provided in figure 4.

The sufficient and necessary condition for equilibrium is $\delta W_{i}=\delta W_{e}$, hence we have

4.3 $$\omega \frac{dA}{d\rho }=l.$$

For rigid origami, where the error $\varepsilon (\rho^{\prime })$ is zero,

4.4 $$\delta W_{e}=l\rho^{\prime }=\delta W_{i}=0.$$

For zero load, the *self-stress* $\omega_{s}$ satisfies

4.5 $$\omega_{s}\frac{dA}{d\rho }=0.$$

Physically, the internal forces could be interpreted as the resistance to deformation around each vertex or cycle, and can be revealed only by cutting through the rigid panels.

**Remark 4.1.**

In rigidity theory, ω is usually referred to as a stress, but we will not use that notation here because of the potential for confusion with the related but different use of the term stress in mechanics. However, we will still use the term self-stress for $\omega_{s}$; a set of internal forces in equilibrium with zero applied load.

Table 1 shows the correspondence between a bar-joint framework and the model for rigid origami considered here. From equation (4.3) and the structure of rigidity matrix dA/dρ, the statement of equilibrium for an inner crease incident to a vertex is that the projection of internal forces on the crease must be equal to the load applied. Consider a vertex; an internal force is a torque $\left\{M_{1},M_{2},M_{3}\right\}$ with three components in a global coordinate system. For each inner crease $j\;(1\leq j\leq N_{\rho })$ with direction vector $p_{j}$,

4.6 $$\left[\begin{array}{ccc} M_{1} & M_{2} & M_{3} \end{array}\right]p_{j}=l_{j}.$$

For an inner crease incident to a cycle, forces also contribute to the equilibrium. For a cycle, the internal force $\left\{M_{1},M_{2},M_{3},F_{1},F_{2},F_{3}\right\}$ has six components of torque and force, which satisfies

4.7 $$\left[\begin{array}{ccc} M_{1} & M_{2} & M_{3} \end{array}\right]p_{j}+\left[\begin{array}{ccc} F_{1} & F_{2} & F_{3} \end{array}\right](O_{j}\times p_{j})=l_{j},$$

which shows that the torque equilibrium is actually

4.8 $$\left(\left[\begin{array}{ccc} M_{1} & M_{2} & M_{3} \end{array}\right]+\left[\begin{array}{ccc} F_{1} & F_{2} & F_{3} \end{array}\right]\times O_{j}\right)p_{j}=l_{j}.$$

**Table 1.** A comparison of the statics of bar-joint framework and rigid origami. In rigid origami, we consider that a ‘body’ is an inner crease with only 1 freedom and the constraint is on each ‘linkage’ of the rigid origami, specifically, each vertex or cycle.

	bar-joint framework	rigid origami
body	joint	inner crease
number of freedoms of a body	3	1
constraints on freedoms	bars	vertices and cycles
form of the internal force	force/length	torque and force
form of the external load	forces	equal and opposite torques

Now that we have clarified the form of the internal forces and load, we can consider static rigidity and its relation to first-order rigidity:

**Definition 4.2.**

A rigid origami can *resolve* a load l if there is an internal force satisfying equation (4.3). A rigid origami is *statically rigid* if it can resolve every load. A rigid origami is *independent* if there is only zero self-stress. A rigid origami is *isostatic* if first-order rigid and independent.

**Theorem 4.3.**

*For a rigid origami* (ρ,λ) *with* $N_{v}$ *inner vertices*, $N_{c}$ *cycles and* $N_{\rho }$ *inner creases, the following statements are equivalent*:

(1) (ρ,λ) *is first-order rigid*.
(2) (ρ,λ) *is statically rigid*.
(3) *The dimension of the self-stress space at* (ρ,λ) *is* $3N_{v}+6N_{c}-N_{\rho }$.

**Proof.**

For the rigidity matrix dA/dρ, a zero nullspace is equivalent to a full image. The rank of its left nullspace when the nullspace is zero is $3N_{v}+6N_{c}-N_{\rho }$.

Examples showing the calculation of internal forces and states of self-stress are given in S3.2 of the electronic supplementary material.

**Remark 4.4.**

We want to comment on the behaviour of a rigid origami under a general load, which might include a set of self-equilibrating forces and torques applied anywhere. We conclude that there would be a unique decomposition of a general load to the form of load discussed in this chapter, i.e. opposite torques applied on adjacent panels around each inner crease. The rest of a general load could always be carried by a rigid origami. A possible way to examine this is to replace the rigid origami by a corresponding double-coning bar-joint framework. Such a framework is generated by replacing the boundary of each panel by a series of bars and joints, then adding two out-of-plane vertices on different sides of the panel, and joining each of the two vertices to each vertex on the panel with a bar.

**Remark 4.5.**

From Dehn’s result [13] on Cauchy’s rigidity theory [14], a strictly convex polyhedral surface is first-order rigid. [15,16] conducted generic rigidity analysis for a *block and hole polyhedral framework*, generated by adding and removing bars to become blocks and holes on a triangulated spherical bar-joint framework. A block and hole polyhedral framework could be realized as a rigid origami. Despite using different models, the rigidity results should be in parallel.

## 5. Prestress stability

In this section, we consider rigid origami that are not first-order rigid but are rigid, and we elucidate how the stability of these structures is changed when prestress is added. To do that, we will describe an energy function U that gives the potential energy stored in the paper. In fact, for our purposes the energy function can be fairly general in its form, but it can also be given in a quite physical way. We will see that the first differential of U with respect to the folding angles naturally gives a state of self-stress for the paper, and the second differential naturally leads to the stiffness and hence stability.

### (a) Energy, stiffness and stability

**Definition 5.1.**

The potential energy U stored in a rigid origami only depends on the error of independent consistency constraint around the $N_{v}$ inner vertices and $N_{c}$ cycles, $A\in R^{3N_{v}+6N_{c}}$, and satisfies

5.1 U(0)=0,U(A)>0 if A≠0.

We require U to have continuous second-order derivative, so that we can define the matrix B as

5.2 $$B=\frac{d^{2}U}{dA^{2}},\;B_{il}=\frac{\partial^{2}U}{\partial A_{i}\;\partial A_{l}}.$$

The size of B is $\left(3N_{v}+6N_{c})\times (3N_{v}+6N_{c}\right)$, which is assumed to be positive-definite. (Note that we are using i and l as subscripts corresponding to error components; later j and k will be used as subscripts for folding angles.)

**Remark 5.2.**

We could consider a less general energy function than that given in equation (5.2), where the energy is the sum of the energy stored around each inner vertex or cycle, in which case the matrix B will be block-diagonal, with one block per inner vertex or cycle. Or we might wish to consider that the energy stored by each misfit error $A_{i}$ is independent, so that B is diagonal. For a particularly simple choice, we could define $E=\sum \frac{1}{2}g_{i}A_{i}^{2}$, so that B would be diagonal and constant, with $B_{ii}=g_{i}$. All of these choices might impact the physical behaviour of the system under load but will not affect the definition of prestress stiffness below.

Next, we will consider the equilibrium of a rigid origami from an energy viewpoint and judge whether it is stable. In general, suppose the rigid origami is in a conservative force field with potential V(ρ), then the total energy could be written as

5.3 E=U+V.

The partial derivative of E with respect to a folding angle $\rho_{j}$ is ($1\leq i\leq 3N_{v}+6N_{c}$, $1\leq j\leq N_{\rho }$)

5.4 $$\frac{\partial E}{\partial \rho_{j}}=\frac{\partial U}{\partial A_{i}}\frac{\partial A_{i}}{\partial \rho_{j}}+\frac{\partial V}{\partial \rho_{j}},$$

which can be written in a more compact form,

5.5 $$\frac{dE}{d\rho }=\frac{dU}{dA}\frac{dA}{d\rho }+\frac{dV}{d\rho }.$$

The equilibrium condition is then

5.6 $$\frac{dU}{dA}\frac{dA}{d\rho }+\frac{dV}{d\rho }=0.$$

Since

5.7 $$l=-\frac{dV}{d\rho },$$

the above condition is exactly equation (4.3), which shows that the first-order derivative of the energy function U is an internal force ω. When dV/dρ=0, the first-order derivative of U is a self-stress.

To consider stability of the equilibrium, we have to consider the second differential, the Hessian of energy, ($1\leq i,l\leq 3N_{v}+6N_{c},1\leq j,k\leq N_{\rho }$)

5.8 $$\frac{\partial^{2}E}{\partial \rho_{k}\partial \rho_{j}}=\frac{\partial^{2}U}{\partial A_{l}\partial A_{i}}\frac{\partial A_{l}}{\partial \rho_{k}}\frac{\partial A_{i}}{\partial \rho_{j}}+\frac{\partial U}{\partial A_{i}}\frac{\partial^{2}A_{i}}{\partial \rho_{k}\partial \rho_{j}}+\frac{\partial^{2}V}{\partial \rho_{k}\partial \rho_{j}},$$

which can be written in a compact form,

5.9 $$\frac{d^{2}E}{d\rho^{2}}={\frac{dA}{d\rho }}^{T}\frac{d^{2}U}{dA^{2}}\frac{dA}{d\rho }+\frac{dU}{dA}\frac{d^{2}A}{d\rho^{2}}-\frac{dl}{d\rho }.$$

The condition for stability is that the total energy at a rigidly folded state reaches a strict local minimum, and a sufficient condition is the second-order differential of the total energy is positive-definite. The second-order derivative $d^{2}A/d\rho^{2}$ is also called the Hessian of the independent consistency constraint A(ρ), an order 3 tensor with size $(3N_{v}+6N_{c})\times N_{\rho }\times N_{\rho }$, which could be written in an explicit form as provided in next subsection.

If there is a perturbation of folding angle δρ around a rigidly folded state (ρ,λ), the increase of total potential energy in the second order will be

5.10 $$\delta E=\frac{1}{2}\delta \rho^{T}\frac{d^{2}E}{d\rho^{2}}\delta \rho ,$$

and the restoring force will be

5.11 $$F=-\frac{\partial \delta E}{\partial \delta \rho }=-\frac{d^{2}E}{d\rho^{2}}\delta \rho .$$

The above derivation shows how $d^{2}E/d\rho^{2}$ works as the stiffness of the rigid origami system. However, if δE=0 for a perturbation δρ, for this direction we might need higher-order information of energy to determine the stability.

In this section, we will discuss the prestress stability first, assuming there is no load (dV/dρ=0).

**Definition 5.3.**

A rigid origami (ρ,λ) with $N_{v}$ inner vertices, $N_{\rho }$ inner creases and $N_{c}$ cycles is *prestress stable* if there is a positive-definite matrix B with size ($3N_{v}+6N_{c})\times (3N_{v}+6N_{c}$) and a vector $\omega_{s}\in R^{3N_{v}+6N_{c}}$ such that

5.12 $$\omega_{s}\frac{dA}{d\rho }=0$$

and

5.13 $$K={\frac{dA}{d\rho }}^{T}B\frac{dA}{d\rho }+\omega_{s}\frac{d^{2}A}{d\rho^{2}}$$

is positive-definite.

Physically, B is the *local elasticity matrix*, which is the Hessian of the predefined energy function. K is the *tangent stiffness matrix* or *total stiffness matrix*. $\omega_{s}\cdot d^{2}A/d\rho^{2}$ is called the *stress matrix*. We say this self-stress $\omega_{s}$ *stabilizes* a rigid origami if it leads to a positive-definite stiffness K.

### (b) Hessian for a vertex or cycle

We will show how to derive the Hessian $d^{2}A/d\rho^{2}$ for a large rigid origami followed by writing $d^{2}A/d\rho^{2}$ for its restriction on a degree-n vertex or cycle. Consider the second-order derivative of equations (2.3) and (2.4).

When 1≤k≤j≤n,

5.14 $$\frac{\partial^{2}}{\partial \rho_{k}\partial \rho_{j}}\left[\begin{array}{c} A_{1}\\ A_{2}\\ A_{3} \end{array}\right]=\left[\begin{array}{c} x_{2k}x_{3j}\\ x_{3k}x_{1j}\\ x_{1k}x_{2j} \end{array}\right],$$

where $x_{j}=[x_{1j};x_{2j};x_{3j}]$ is the direction vector of the inner crease $\rho_{j}$ measured in a global coordinate system, pointing away from this vertex.

5.15 $$\frac{\partial^{2}}{\partial \rho_{k}\partial \rho_{j}}\left[\begin{array}{c} A_{1}\\ A_{2}\\ A_{3}\\ A_{4}\\ A_{5}\\ A_{6} \end{array}\right]=\left[\begin{array}{c} x_{2k}x_{3j}\\ x_{3k}x_{1j}\\ x_{1k}x_{2j}\\ x_{k}\times (O_{j}\times x_{j}) \end{array}\right],$$

where $x_{j}=[x_{1j};x_{2j};x_{3j}]$ is the direction vector of the inner crease $\rho_{j}$ measured in a global coordinate system, pointing away from this cycle. $O_{j}$ is the position of vertex on the cycle incident to $\rho_{j}$ measured in the global coordinate system (figure 2). When 1≤j<k≤n, swap k and j. The derivation is provided in S2 of the electronic supplementary material.

### (c) Hessian for a large rigid origami

Now we consider assembling the second-order derivative for each vertex or cycle in equations (5.14) and (5.15) to a large rigid origami. The Hessian $d^{2}A/d\rho^{2}$ is an order 3 tensor with size $(3N_{v}+6N_{c})\times N_{\rho }\times N_{\rho }$, which is the collection of sparse matrices $d^{2}A_{i}/d\rho^{2}$. The size of each of them is $N_{\rho }\times N_{\rho }$.

Consider the incidence matrix for vertices $D_{\mathrm{vertex}}$; row $i(1\leq i\leq N_{v})$ corresponds with three components $\left[A_{3i-2};A_{3i-1};A_{3i}\right]$. For folding angle $\rho_{j}$ where the direction vector is $p_{j}$ in a global coordinate system, if $D_{ij}=1$, $x_{j}=p_{j}$, otherwise if $D_{ij}=-1$, $x_{j}=-p_{j}$, then by applying equation (5.14) we could obtain $3N_{v}$ matrices $d^{2}A_{i}/d\rho^{2}$ for every vertex.

Next consider the incidence matrix for cycles $D_{\mathrm{cycle}}$; row $i(1\leq i\leq N_{c})$ corresponds with six components $\left[A_{3N_{v}+6i-5};A_{3N_{v}+6i-4};A_{3N_{v}+6i-3};A_{3N_{v}+6i-2};A_{3N_{v}+6i-1};A_{3N_{v}+6i}\right]$. For folding angle $\rho_{j}$, if $D_{ij}=1$, $x_{j}=p_{j}$, otherwise if $D_{ij}=-1$, $x_{j}=-p_{j}$, then by applying equation (5.15) we could obtain $6N_{c}$ matrices $d^{2}A_{i}/d\rho^{2}$ for every cycle.

### (d) Reducing the calculation

From definition 5.3, if a rigid origami is first-order rigid-foldable and there is no prestress applied, it is not stable. Hence, for a given configuration, an important question is to find the space of self-stress to stabilize a rigid origami. The next proposition provides a simpler way to judge the prestress stability.

**Proposition 5.4.**

*Some statements concerning the positive (semi)-definiteness of the stiffness matrix for a first-order rigid-foldable rigid origami*:

(1) *The matrix* $\left[{\left(dA/d\rho \right)}^{T}\cdot B\cdot dA/d\rho \right]$ *is positive semi-definite. Both of its nullspace and roots of quadratic form are the nullspace of* dA/dρ.
(2) *A rigid origami* (ρ,λ) *is prestress stable if, and only if, there exists a self-stress* $\omega_{s}\in R^{3N_{v}+6N_{c}}$ *such that* $\omega_{s}\cdot d^{2}A/d\rho^{2}$ *is positive-definite when restricted to the nullspace of* dA/dρ.
(3) *A rigid origami* (ρ,λ) *is prestress stable if, and only if, there exists a self-stress* $\omega_{s}\in R^{3N_{v}+6N_{c}}$ *such that all the eigenvalues of* ${\left[\rho^{\prime }\right]}^{T}\cdot \omega_{s}\cdot d^{2}A/d\rho^{2}\cdot [\rho^{\prime }]$ *are positive, where* $\left[\rho^{\prime }\right]$ *is a basis of the space of first-order flex*.
  5.16 $$[\rho^{\prime }]=\left[\begin{array}{cccc} \rho_{1}^{\prime } & \rho_{2}^{\prime } & \cdots & \rho_{N_{\rho }-\mathrm{rank}(dA/d\rho )}^{\prime } \end{array}\right].$$

**Proof.**

Statement (1): For any perturbation of folding angles δρ, consider the quadratic form; if δρ is not a first-order flex, since B is assumed to be positive-definite, $\delta \rho^{T}\cdot {\left(dA/d\rho \right)}^{T}\cdot B\cdot dA/d\rho \cdot \delta \rho >0$, therefore $\left[{\left(dA/d\rho \right)}^{T}\cdot B\cdot dA/d\rho \right]$ is positive semi-definite, and the roots of quadratic form of $\left[{\left(dA/d\rho \right)}^{T}\cdot B\cdot dA/d\rho \right]$ are contained in the nullspace of dA/dρ. Since the nullspace of dA/dρ is also contained in the roots of quadratic form of $\left[{\left(dA/d\rho \right)}^{T}\cdot B\cdot dA/d\rho \right]$, the statement holds. Then consider the linear form. For any δρ, if ${\left(dA/d\rho \right)}^{T}\cdot B\cdot dA/d\rho \cdot \delta \rho =0$, $\delta \rho^{T}\cdot {\left(dA/d\rho \right)}^{T}\cdot B\cdot dA/d\rho \cdot \delta \rho =0$, therefore the linear nullspace is contained in the roots of quadratic form. If δρ is in the roots of quadratic form, it is a first-order flex, therefore being an element of the linear nullspace, hence the statement holds.

Statement (2): Necessity: if a rigid origami is prestress stable, the quadratic form of a first-order flex should be greater than 0; hence $\omega_{s}\cdot d^{2}A/d\rho^{2}$ is positive-definite when restricted to the nullspace of dA/dρ.

Sufficiency: We will show that, if there exists a self-stress $\omega_{s}$ such that $\omega_{s}\cdot d^{2}A/d\rho^{2}$ is positive-definite when restricted to the nullspace of dA/dρ, by choosing a sufficiently large k, $K={\left(dA/d\rho \right)}^{T}\cdot B\cdot dA/d\rho +k\omega_{s}\cdot d^{2}A/d\rho^{2}$ would be positive-definite.

For any perturbation of folding angles δρ, if δρ is a first-order flex, for any k>0, $\delta \rho^{T}K\delta \rho >0$. If δρ is not a first-order flex, suppose ||δρ||=1. Since this set is compact, there exists ε>0 s.t. $\delta \rho^{T}\cdot {\left(dA/d\rho \right)}^{T}\cdot B\cdot dA/d\rho \cdot \rho \geq \varepsilon$ and we know $\delta \rho^{T}\cdot \omega_{s}\cdot d^{2}A/d\rho^{2}\cdot \delta \rho \geq -||\omega_{s}\cdot d^{2}A/d\rho^{2}||$, then we can choose $0<k<\varepsilon /||\omega_{s}\cdot d^{2}A/d\rho^{2}||$ s.t. $\delta \rho^{T}K\delta \rho >0$. Furthermore, consider ||δρ||≠1; choosing the same k, $\delta \rho^{T}K\delta \rho /||\delta \rho |{|}^{2}>0$.

Statement (3): Since a first-order flex $\rho^{\prime }$ could be written as

5.17 $$\rho^{\prime }=[\rho^{\prime }]a=\left[\begin{array}{cccc} \rho_{1}^{\prime } & \rho_{2}^{\prime } & \cdots & \rho_{N_{\rho }-\mathrm{rank}(dA/d\rho )}^{\prime } \end{array}\right]\left[\begin{array}{c} a_{1}\\ a_{2}\\ \vdots \\ a_{N_{\rho }-\mathrm{rank}(dA/d\rho )} \end{array}\right],$$

this statement will hold from statement (2).

Calculation of specific examples that are prestress stable but not first-order rigid are provided in S3.3 of the electronic supplementary material. There is a general result for a rigid and planar *single-vertex*, which refers to a rigid origami with only one inner vertex and no cycles. The proof is given in S4 of the electronic supplementary material.

**Proposition 5.5.**

*A rigid planar single-vertex is prestress stable.*

Furthermore, any triangulated convex polyhedral surface (possibly with some properly placed holes) is prestress stable [17]. This is drawn from the analysis of a ‘spider tensegrity’ and would be applicable to rigid origami.

### (e) Considering external load

When there is a load l(ρ) applied on a rigid origami, the above theory on stability could be modified as below.

**Definition 5.6.**

A rigid origami (ρ,λ) with $N_{v}$ inner vertices, $N_{\rho }$ inner creases and $N_{c}$ cycles is stable under load l(ρ) if there is a positive-definite matrix B with size ($3N_{v}+6N_{c})\times (3N_{v}+6N_{c}$) and a vector $\omega \in R^{3N_{v}+6N_{c}}$ such that

5.18 $$\omega \frac{dA}{d\rho }=l$$

and

5.19 $$K={\frac{dA}{d\rho }}^{T}B\frac{dA}{d\rho }+\omega \frac{d^{2}A}{d\rho^{2}}-\frac{dl}{d\rho }$$

is positive-definite.

**Proposition 5.7.**

*A rigid origami* (ρ,λ) *is stable under load* l(ρ) *if, and only if, there exists a stress* $\omega \in R^{3N_{v}+6N_{c}}$ *such that* $\omega \cdot d^{2}A/d\rho^{2}$ *is positive-definite when restricted to the nullspace of* dA/dρ. *Equivalently, a rigid origami is stable if, and only if, there exists a stress* $\omega \in R^{3N_{v}+6N_{c}}$ *such that all the eigenvalues of* ${\left[\rho^{\prime }\right]}^{T}\cdot \omega \cdot d^{2}A/d\rho^{2}\cdot [\rho^{\prime }]$ *are positive. Here* $\left[\rho^{\prime }\right]$ *is a basis of the space of first-order flex in equation* (5.16).

**Proof.**

From statement (2) of proposition 5.4, (ρ,λ) is stable if, and only if, there exists a stress $\omega \in R^{3N_{v}+6N_{c}}$ such that $\omega \cdot d^{2}A/d\rho^{2}-dl/d\rho$ is positive-definite when restricted to the nullspace of dA/dρ. Since a first-order flex $\rho^{\prime }$ is orthogonal to l, the quadratic form $\rho^{\prime }\cdot dl/d\rho \cdot \rho =0$.

**Remark 5.8.**

In equation (5.19), the first term ${\left(dA/d\rho \right)}^{T}\cdot B\cdot dA/d\rho$ could be interpreted as the material part of the stiffness matrix, which only relates to how the potential energy stored in a rigid origami is defined, and is assumed to be semi positive-definite with nullspace as the space of first-order flex. The second term $\omega \cdot d^{2}A/d\rho^{2}$ shows how a load could possibly enhance or reduce the stiffness. Furthermore, the third term −dl/dρ will change the restoring force but has no effect on the stability.

An example showing how load would affect the stability is also given in S3.3 of the electronic supplementary material.

## 6. Second-order rigidity

In this section, we will discuss the second-order rigidity and show its link with prestress stability. For a rigid origami (ρ,λ), a first-order flex $\rho^{\prime }$ is obtained by differentiating the independent consistency constraint A(ρ)=0. Similarly, a second-order flex $\left(\rho^{\prime },\rho^{\prime \prime }\right)$ satisfies the condition from differentiating the consistency constraint twice.

**Definition 6.1.**

For a rigid origami (ρ,λ) with $N_{v}$ inner vertices, $N_{\rho }$ inner creases and $N_{c}$ cycles, a *second-order flex* $\left(\rho^{\prime },\rho^{\prime \prime })\in (R^{N_{\rho }},R^{N_{\rho }}\right)$ satisfies ($1\leq i\leq 3N_{v}+6N_{c},1\leq j,k\leq N_{\rho }$)

6.1 $$\begin{array}{rl} & \frac{\partial A_{i}}{\partial \rho_{j}}\rho_{j}^{\prime }=0\\ \mathrm{and}\;\; & \frac{\partial^{2}A_{i}}{\partial \rho_{j}\partial \rho_{k}}\rho_{j}^{\prime }\rho_{k}^{\prime }+\frac{\partial A_{i}}{\partial \rho_{j}}\rho_{j}^{\prime \prime }=0. \end{array}\}$$

If written in a compact form,

6.2 $$\begin{array}{rl} & \frac{dA}{d\rho }\rho^{\prime }=0\\ \mathrm{and}\;\; & \rho^{\prime T}\frac{d^{2}A}{d\rho^{2}}\rho^{\prime }+\frac{dA}{d\rho }\rho^{\prime \prime }=0. \end{array}\}$$

A second-order flex with $\rho^{\prime }=0$ is called *trivial*, or otherwise *non-trivial*. If there is only trivial second-order flex, this rigid origami is *second-order rigid*, or otherwise *second-order rigid-foldable*.

**Proposition 6.2.**

*Some statements concerning the second-order rigidity and prestress stability*:

(1) *If* $\left(\rho^{\prime },\rho^{\prime \prime }\right)$ *is a second-order flex and* $\rho_{0}^{\prime }$ *is a first-order flex*, $\left(\rho^{\prime },\rho^{\prime \prime }+\rho_{0}^{\prime }\right)$ *is also a second-order flex*.
(2) *A first-order flex* $\rho^{\prime }$ *can be extended to a second-order flex* $\rho^{\prime \prime }$ *if, and only if, for all self-stress* $\omega_{s}$, $\rho^{\prime T}[\omega_{s}\cdot d^{2}A/d\rho^{2}]\;\rho^{\prime }=0$.
(3) *A rigid origami is second-order rigid if, and only if, for any first-order flex* $\rho^{\prime }$ *there is a self-stress* $\omega_{s}(\rho^{\prime })$ *s.t.*, $\rho^{\prime T}[\omega_{s}\cdot d^{2}A/d\rho^{2}]\;\rho^{\prime }>0$.
(4) *A rigid origami is second-order rigid if, and only if, the intersection of roots of quadratic form of all* ${\left[\rho^{\prime }\right]}^{T}\cdot \omega_{i}\cdot d^{2}A/d\rho^{2}\cdot [\rho^{\prime }]$ *is* 0. *Here* $\left\{\omega_{i}\right\}$ *is a basis of self-stress* $\left(1\leq i\leq 3N_{v}+6N_{c}-\mathrm{rank}(dA/d\rho )\right)$.
(5) *A rigid origami is prestress stable if second-order rigid and* $\mathrm{rank}(dA/d\rho )=N_{\rho }-1$ *or* $\mathrm{rank}(dA/d\rho )=3N_{v}+6N_{c}-1$.

**Proof.**

Statement (1): This can be verified directly from definition 6.1.
Statement (2): A first-order flex can be extended to a second-order flex if, and only if, there exists a solution for the linear system below
  6.3 $$\frac{dA}{d\rho }\rho^{\prime \prime }=-\rho^{\prime T}\frac{d^{2}A}{d\rho^{2}}\rho^{\prime },$$
  i.e. that the vector $\left(\rho^{\prime T}\cdot d^{2}A/d\rho^{2}\cdot \rho^{\prime }\right)$ lies in the column space of the matrix dA/dρ. Any self-stress $\omega_{s}$ lies in the left nullspace (the orthogonal complement of the column space) of dA/dρ, and hence $\omega_{s}(\rho^{\prime T}\cdot d^{2}A/d\rho^{2}\cdot \rho^{\prime })=0$. The order of the first two terms in the expression can be swapped without affecting the outcome, and hence the statement is proved.
Statement (3): We know from the inverse negative of statement (2) that a rigid origami is second-order rigid if, and only if, for any first order-flex $\rho^{\prime }$ there is a self-stress $\omega_{s}(\rho^{\prime })$ such that $\rho^{\prime T}[\omega_{s}\cdot d^{2}A/d\rho^{2}]\;\rho^{\prime }\neq 0$. Either this quadratic form is positive, or it can be made positive by replacing $\omega_{s}$ with $-\omega_{s}$.
Statement (4): If a first-order flex $\rho^{\prime }=[\rho^{\prime }]a$ can be extended to a second-order flex, a should be a quadratic root for every ${\left[\rho^{\prime }\right]}^{T}\cdot \omega_{i}\cdot d^{2}A/d\rho^{2}\cdot [\rho^{\prime }]$ such that $a^{T}{\left[\rho^{\prime }\right]}^{T}\cdot \omega_{i}\cdot d^{2}A/d\rho^{2}\cdot [\rho^{\prime }]a=0$, which leads to this statement.
Statement (5): From statement (3), for any first-order flex $\rho^{\prime }$ there is a self-stress $\omega_{s}(\rho^{\prime })$ such that $\rho^{\prime T}[\omega_{s}\cdot d^{2}A/d\rho^{2}]\;\rho^{\prime }>0$. If $\mathrm{rank}(dA/d\rho )=N_{\rho }-1$, the dimension of the space of first-order flex is 1 and the nullspace of dA/dρ is $c\rho_{1}^{\prime },c\in R$. The self-stress $\omega_{s}(\rho_{1}^{\prime })$ will stabilize this rigid origami since $\rho^{\prime T}[\omega_{s}(\rho_{1}^{\prime })\cdot d^{2}A/d\rho^{2}]\;\rho^{\prime }=c^{2}\rho_{1}^{\prime T}[\omega_{s}(\rho_{1}^{\prime })\cdot d^{2}A/d\rho^{2}]\;\rho_{1}^{\prime }>0$. Next, if the dimension of the space of self-stress is 1, denote this basis vector as $\omega_{1}$. If this rigid origami is not prestress stable, there will exist a first-order flex $\rho^{\prime }$ such that for all choices of c, $c\rho^{\prime T}[\omega_{1}\cdot d^{2}A/d\rho^{2}]\;\rho^{\prime }\leq 0$. First, $\rho^{\prime T}[\omega_{1}\cdot d^{2}A/d\rho^{2}]\;\rho^{\prime }\neq 0$ since the rigid origami is second-order rigid. Second, by choosing c=±1, $c\rho^{\prime T}[\omega_{1}\cdot d^{2}A/d\rho^{2}]\;\rho^{\prime }$ could be greater than 0. These lead to a contradiction.

**Remark 6.3.**

Prestress stability requires a single self-stress ω such that the quadratic form is positive for every first-order flex, while the second-order rigidity requires a ‘suitable’ self-stress $\omega (\rho^{\prime })$ for every first-order flex such that the quadratic form is positive. Physically, such a $\omega (\rho^{\prime })$ ‘blocks’ a possible second-order flex for a given first-order flex.

**Remark 6.4.**

For a planar rigid origami, after defining a reciprocal diagram, the first- and second-order rigid-foldability could be graphically explained as the existence, and the zero-area property, of the reciprocal diagram [18–20].

An attempt to find a rigid origami that is second-order rigid but not prestress stable is provided in S3.4 of the electronic supplementary material. Similar to a single-vertex in proposition 5.5, a *single-hole* means a rigid origami with only one cycle and no inner vertices. Here, we conjecture that (*regular* means the rigidity matrix has maximum rank):

**Conjecture 6.5.**

A rigid but not regular single-hole is prestress stable.

## 7. Relation among different levels of rigidity

In this section, we will prove the relation among the rigidity discussed in the above sections and which is illustrated in figure 1.

**Theorem 7.1.**

*The relation among first-order or static rigidity, prestress stability and second-order rigidity.*

(1) *A rigid origami is prestress stable if first-order rigid or statically rigid.*
(2) *A rigid origami is second-order rigid if prestress stable.*
(3) *A rigid origami is rigid if second-order rigid.*

**Proof.**

Statement (1): Set $\omega_{s}=0$; the total stiffness $K={\left(dA/d\rho \right)}^{T}\cdot B\cdot dA/d\rho$ is now positive-definite.
Statement (2): If a rigid origami is prestress stable, for any first-order flex $\rho^{\prime }$ there is a uniform $\omega_{s}$ such that $\rho^{\prime T}[\omega_{s}\cdot d^{2}A/d\rho^{2}]\;\rho^{\prime }>0$. From statement (3) of proposition 6.2, this rigid origami is second-order rigid.
Statement (3): We need to prove that for rigid origami, a continuous flex implies a second-order flex. This could be done by transferring the consistency constraint A(ρ)=0 to a polynomial system A(t)=0 with the normalized folding angle description t=tan⁡(ρ/2), and we claim that the definitions on local rigidity are equivalent for these two expressions (details are provided in S5 of the electronic supplementary material). It turns out that a continuous flex is equivalent to an analytical flex in the normalized folding angle description [8, §4]. Denote an analytical flex for a foldable rigid origami starting from (t,λ) by γ:[0,1]∋s→{t}. This flex could be parametrized by a single s
  7.1 $$\gamma =t+\sum_{n=1}^{\infty }\frac{a_{n}}{n!}s^{n},\;A(\gamma )\equiv 0,$$
  where not all $a_{n}=0$.

If $a_{1}\neq 0$, as we know that

7.2 $$\frac{dA}{ds}{|}_{s=0}=0,\;\frac{d^{2}A}{ds^{2}}{|}_{s=0}=0$$

then $\left(a_{1},a_{2}\right)$ would be a second-order flex satisfying equation (5.10).

If $a_{1}=0$, then as γ≠0, there must be some first non-zero term $a_{k}$. Then, as we know that

7.3 $$\frac{d^{i}A}{ds^{i}}{|}_{s=0}=0,\;1\leq i\leq 2k,$$

hence $a_{k}$ would be a first-order flex, and (ak,2a2k/(2kk)) would be a second-order flex satisfying equation (5.10). Here, (2kk) is the binomial coefficient.

Note that none of the statements in theorem 7.1 are reversible. In S3.3 and S3.4 of the electronic supplementary material, we show examples that are prestress stable but not first-order rigid. In S3.5 of the electronic supplementary material, we show an example that is rigid but not second-order rigid.

## 8. From local rigid-foldability to rigid-foldability

When studying the hierarchical relation described in theorem 7.1, it turns out that for some rigid origami, different levels of rigidity might be equivalent. In particular, a first-order flex which will not lead to crossing of panels might be extended to a continuous flex.

**Proposition 8.1.**

*Extension of local rigid-foldability for some special rigid origami. Recall that a rigid origami is regular if the rigidity matrix has maximum rank.*

(1) *A regular rigid origami is rigid-foldable (allowing self-intersection) if first-order rigid-foldable*.
(2) *A single-vertex is rigid-foldable (allowing self-intersection) if not prestress stable.*
(3) *A planar quadrilateral mesh where each vertex is flat-foldable is at least second-order rigid-foldable.*

**Proof.**

Statement (1): Suppose there are $N_{v}$ inner vertices, $N_{c}$ cycles and $N_{\rho }$ inner creases. From the Implicit Function Theorem [21], §8.5, at a neighbourhood of ρ, the folding angle space would be a manifold with dimension $N_{\rho }-\mathrm{rank}(dA/d\rho )=N_{\rho }-3N_{v}-6N_{c}>0$, hence there would be a continuous flex starting from (ρ,λ).

Statement (2): For a non-planar single-vertex, it is regular, hence either first-order rigid or rigid-foldable. For a planar single-vertex, from proposition 5.5, if not prestress stable it would be rigid-foldable.

Statement (3): If each vertex of a planar quadrilateral mesh is flat-foldable, the relations among the tangent of half of all the folding angles, shown in the consistency constraint, are linear, even though this quadrilateral mesh might not be rigid-foldable [22]. Consider the normalized folding angle description; the consistency constraint A(t)=0 could be rewritten as a linear system $A^{\prime }(t)=0$ among t, hence the Hessian $d^{2}A^{\prime }/dt^{2}$ is zero. Since a planar quadrilateral mesh is also first-order rigid-foldable, it cannot be prestress stable or second-order rigid.

A rigid but not second-order rigid example for statement (3) of proposition 8.1 is provided in S3.5 of the electronic supplementary material, but the stress matrix $\omega_{s}\cdot d^{2}A/d\rho^{2}$ is not zero. An explanation is that although A(t)=0 is essentially a linear system with the special choice of sector angles such that every vertex is flat-foldable, it would be in the form of a complicated polynomial system consisting the square of linear relations, hence $\omega_{s}\cdot d^{2}A/d\rho^{2}$ is not zero. Furthermore, not every first-order flex can be extended to a second-order flex in this example when choosing the consistency constraint to be A(ρ)=0. We claim that the conclusion on local rigidity should be invariant to the choice of form of consistency constraint.

Proposition 8.1 opens a promising topic for a rigid origami; that is, to explore the level of local rigidity and find whether some of these levels are in fact equivalent.

As stated in the introduction, when some folding angles are ±π, a first-order flex calculated from the independent consistency constraint A(ρ)=0 is extendable to a flex only when this first-order flex induces angle change within [−π,π]. Examples on this topic are provided in S3.6 of the electronic supplementary material.

## 9. Numerical methods for rigidity analysis

In this section, we will consider how to analyse the local rigidity for a rigid origami using numerical methods when the size of dA/dρ is large. Several important questions are as follows:

(1) How do we determine the first-order rigidity of a rigid origami, or find the space of first-order flex and self-stress?
(2) Will a given self-stress stabilize a rigid origami?
(3) How do we find the space of self-stress that can stabilize a rigid origami?
(4) Can a first-order flex be extended to a second-order flex?
(5) How do we find the space of first-order flex that can be extended to a second-order flex?

For question (1), if we know the position of each vertex, the direction vector of each inner crease can be calculated directly, and the rigidity matrix dA/dρ in a global coordinate system can be obtained by assigning entries for a sparse matrix. The next step is applying the singular value decomposition to dA/dρ to find the information of rank, nullspace and left nullspace [23,24].

Question (2) is the forward problem of determining prestress stability. The Hessian $d^{2}A/d\rho^{2}$ of a rigid origami can also be obtained by assigning entries for a sparse matrix if the position of each vertex is known. With the information of the nullspace of dA/dρ calculated in (1), we need to know the positive definiteness of ${\left[\rho^{\prime }\right]}^{T}\cdot \omega_{s}\cdot d^{2}A/d\rho^{2}\cdot [\rho^{\prime }]$ from statement (3) of proposition 5.4, which is symmetric. The eigenvalues and eigenvectors for a real, sparse and symmetric matrix could be found, for instance, by the modified Lanczos algorithm [25,26].

Question (3) is the inverse problem of determining prestress stability. The space of self-stress that can stabilize a rigid origami turns out to be an elliptic set. Suppose the basis of self-stress is $\omega_{s}^{i}$ ($1\leq i\leq 3N_{v}+6N_{c}-\mathrm{rank}(dA/d\rho )$), which is calculated in (1). Now the problem becomes one of whether there is a linear combination of these $3N_{v}+6N_{c}-\mathrm{rank}(dA/d\rho )$ real, sparse and symmetric matrices ${\left[\rho^{\prime }\right]}^{T}\cdot \omega_{s}^{i}\cdot d^{2}A/d\rho^{2}\cdot [\rho^{\prime }]$ that is positive-definite. This is a problem in semi-definite programming that has been well-studied [27]. We could set this problem as

$$\begin{array}{rl} & \mathrm{minimize}\;d^{T}c\\ & s.t.\;\sum c_{i}{\left[\rho^{\prime }\right]}^{T}\omega_{s}^{i}\frac{d^{2}A}{d\rho^{2}}[\rho^{\prime }]\;\text{positive-definite, } \end{array}$$

where $d\in R^{n}$ is a given vector that converges the solution set of c to be elliptic. Note that even if a stabilizing self-stress $\omega_{s}$ is found, the proof of existence of k is not constructive in proposition 5.4. Other techniques need to be applied to determine how small k has to be.

Question (4) is the forward problem of determining second-order rigidity. From statement (2) in proposition 6.2, the problem is to consider whether the given first-order flex ρ’ is in the roots of quadratic form of every $\omega_{s}^{i}\cdot d^{2}A/d\rho^{2}$, which is calculated in (3). If not, $\rho^{\prime }$ could be extended to a second-order flex.

Question (5) is the inverse problem of determining second-order rigidity. The space of first-order flex that can be extended to a second-order flex is also an elliptic system. From statement (4) in proposition 6.2, we need to find the common root of the quadratic form for each ${\left[\rho^{\prime }\right]}^{T}\cdot \omega_{s}^{i}\cdot d^{2}A/d\rho^{2}\cdot [\rho^{\prime }]$, where $\left\{\omega_{s}^{i}\right\}$ is a basis of self-stress $\left(1\leq i\leq 3N_{v}+6N_{c}-\mathrm{rank}(dA/d\rho )\right)$. Since each ${\left[\rho^{\prime }\right]}^{T}\cdot \omega_{s}^{i}\cdot d^{2}A/d\rho^{2}\cdot [\rho^{\prime }]$ is real and symmetric, we could write its eigenvalues as $s_{j}$ and its orthonormal vectors as $v_{j},(1\leq j\leq N_{\rho }-\mathrm{rank}(dA/d\rho ))$. If a is a root of the quadratic form,

9.1 $$\begin{array}{rl} & a=\sum_{1}^{N_{\rho }-\mathrm{rank}(dA/d\rho )}c_{j}v_{j}\\ \mathrm{and}\;\; & \;\sum_{1}^{N_{\rho }-\mathrm{rank}(dA/d\rho )}c_{j}^{2}s_{j}=0. \end{array}\}$$

That is to say, the square of coefficients $c^{2}$ when $a=\sum c_{j}v_{j}$ should be orthogonal to the eigenvalues s. The next step is to find the intersection of such $3N_{v}+6N_{c}-\mathrm{rank}(dA/d\rho )$ solution space of quadratic form for each basis vector of self-stress. For a large rigid origami, the computation would be expensive.

## 10. Conclusion

We have shown that rigid origami can be advantageously analysed from a rigidity point of view. This is an inversion of the usual focus on folding. Rather than considering when a paper can be folded, we have examined various ways in which the design of an origami might prevent folding. This article is a strong complement to classic rigidity theory with the rigid origami model, where new forms of constraint, internal force and geometric error are elucidated. We think this perspective will prove to be of further use in the development of novel folding patterns, or indeed in the design of structures formed from origami where some rigidity is required.

## Data accessibility

All scripts used in this study are openly accessible through https://github.com/StochasticBiology/boolean-efflux.git. The data are provided in electronic supplementary material [28].
